# A simulated night shift in the emergency room increases students’ self-efficacy independent of role taking over during simulation

**DOI:** 10.1186/s12909-016-0699-9

**Published:** 2016-07-15

**Authors:** Fabian Stroben, Therese Schröder, Katja A. Dannenberg, Anke Thomas, Aristomenis Exadaktylos, Wolf E. Hautz

**Affiliations:** Lernzentrum (Skills Lab), Charité-Universitätsmedizin Berlin, Charitéplatz 1, 10117 Berlin, Germany; Department of Gynecology and Obstretics CCM & CVK, Charité-Universitätsmedizin Berlin, Charitéplatz 1, 10117 Berlin, Germany; Department of Emergency Medicine at Campus Benjamin Franklin, Charité-Universitätsmedizin Berlin, Charitéplatz 1, 10117 Berlin, Germany; Universitäres Notfallzentrum, Inselspital Bern, 3010 Bern, Switzerland

**Keywords:** Medical education, Undergraduate education, Simulation-based education, Emergency medicine, High-fidelity simulation, Self-assessment, Self-efficacy

## Abstract

**Background:**

Junior doctors do not feel well prepared when they start into postgraduate training. High self-efficacy however is linked to better clinical performance and may thus improve patient care. What factors affect self-efficacy is currently unknown. We conducted a simulated night shift in an emergency room (ER) with final-year medical students to identify factors contributing to their self-efficacy and thus inform simulation training in the ER.

**Methods:**

We simulated a night in the ER using best educational practice including multi-source feedback, simulated patients and vicarious learning with 30 participants. Students underwent 7 prototypic cases in groups of 5 in different roles (leader, member and observer). Feeling of preparedness was measured at baseline and 5 days after the event. After every case students recorded their confidence dependent of their role during simulation and evaluated the case.

**Results:**

Thirty students participated, 18 (60 %) completed all surveys. At baseline students feel unconfident (Mean −0.34). Feeling of preparedness increases significantly at follow up (Mean 0.66, *p* = 0.001, *d* = 1.86). Confidence after simulation is independent of the role during simulation (F(2,52) = 0.123, *p* = 0.884). Observers in a simulation can estimate leader’s confidence independent of their own (*r* = 0.188, *p* = 0.32) while team members cannot (*r* = 0.61, *p* < 0.001).

**Conclusions:**

Simulation improves self-efficacy. The improvement of self-efficacy is independent of the role taken during simulation. As a consequence, groups can include observers as participants without impairing their increase in self-efficacy, providing a convenient way for educators to increase simulation efficiency. Different roles can furthermore be included into multi-source peer-feedback.

**Electronic supplementary material:**

The online version of this article (doi:10.1186/s12909-016-0699-9) contains supplementary material, which is available to authorized users.

## Background

Junior doctors do not feel well prepared when they start into postgraduate training [1–3] independent of their objective performance [4]. Next to the accuracy of a diagnosis, adequate confidence in this diagnosis however is a necessity for safe and effective patient care. Too little confidence in an accurate diagnosis may harm patients through the delay of necessary treatment and unnecessary and potentially harmful additional investigations.

While the relationship between confidence and tendency to act applies to all of medicine, it is especially relevant to emergency medicine, where delayed action may have severe consequences.

Situational confidence (or self-efficacy) is a key factor to determine what actions one may take [5]. As an individual’s reliance on personal abilities to succeed in a given challenge, self-efficacy increases the likelihood of that individual’s actions actually occurring [6, 7]. By contrast, low self-efficacy and resulting distress is argued to contribute to mental health problems [8, 9].

Several factors have been identified to influence self-reported feelings of preparedness. The percentage of graduates not feeling well prepared for clinical work differs strongly between countries [3, 10, 11] implying a great impact of educational systems and practices. Factors known to contribute to higher feelings of preparedness include frequent and immediate feedback [12], theoretical education counterbalanced with practice training, good skills education and training in diagnostic decision-making [10].

Simulation is a teaching format that may (and should) contain all four of those elements [13, 14] and thus should affect individual feelings of preparedness besides the well-known effects on objective performance [15]. Another teaching format known to increase self-efficacy includes observational or vicarious learning which is as effective as hands-on training in the acquisition of practical skills [16].

The aim of our study was to develop a best practice simulation session and evaluate the effect of simulation on the development of students’ feelings of preparedness. We further aimed to identify factors within the simulation that affect confidence and feelings of preparedness in order to design a well-balanced simulation, budgeting both costs and educational effectiveness. To identify such factors we focused on the role students take over during the simulation and differences between self-reported confidence and confidence judged by peers.

## Methods

### Study design

A six-hour simulation session took place in 2013 at Charité – Universitätsmedizin Berlin as a night shift in a simulated emergency room (ER). The ER consisted of several rooms and an ambulance vehicle. Each room hosted a different simulated case of a total of seven. We invited students in their final year of medical school to participate.

**Fig. 1:**
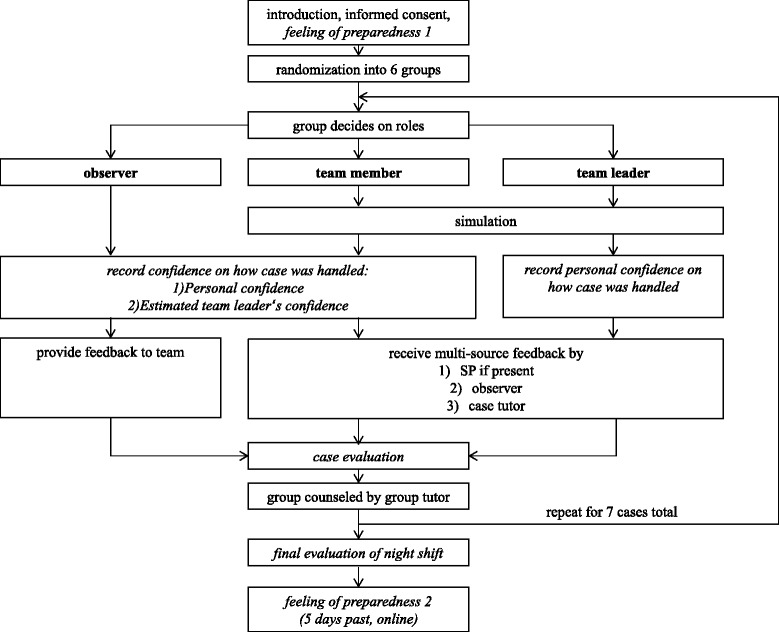
Study design. Measurements in italic, roles in bold

Participants were randomized into teams of five. Each team rotated through each of the scenarios, thus seeing seven different patients, each presenting a typical ER case. Each group was staffed with a peer tutor who counseled on teamwork in between scenarios, helped with logistics and ensured participants filled in evaluations. Each room was staffed with a case tutor who ran the simulation scenario. Before starting each scenario, the group decided on a team leader, team members and observers. Feedback was given after each scenario. Figure 1 illustrates the study design.

At the beginning of the night, participants completed a questionnaire on possible confounder and self-reported feeling of preparedness in different medical specialties together with an informed consent form. Directly after each scenario, all active members recorded their confidence individually before feedback was given. Furthermore team members and observers estimated the team leader's confidence. After feedback, participants evaluated the quality of the given feedback and of the simulation overall. At the end of the night, participants filled in a final evaluation focusing on overall quality of the simulations. Participants were further asked to complete a second questionnaire of self-reported feelings of preparedness five days after the event in an online survey similar to the first questionnaire.

All evaluations (forms available upon request) were conducted using Likert scales for each item ranging from “totally agree” to “totally disagree”. We coded the responses on a numerical scale ranging from +3 to −3 with 0 equating “neutral”. All but the last evaluation forms were filled in on paper during the simulation, the final questionnaire “feeling of preparedness 2” was conducted online using lime survey software.

### Participants

Medical students who had completed their fifth year of medical school (around 600 total) were invited to take part in the night shift. In Germany, five years of medical studies are conducted at university, the sixth and final year is spent in internships at different hospitals. Participants were chosen on a first come-first served basis through an online registration. 30 participants were randomized into six teams stratified by gender using a computer-generated randomization list. Participants were greeted in a general assembly and informed about the course of events of the night. After completing the written informed consent including participant information, information about opportunities to withdraw and possible consequences of withdrawl (none), they split into teams. The study was approved by the institutional office for data protection at Charité Berlin and deemed exempt from ethical review under local legislation, because it does not involve patients.

### Cases

Cases were drafted following national and international guidelines and chosen based on learning objectives from a German national consensus statement [17, 18]. Each case represented a common ER patient. There were more diagnostic investigations available than necessary per case in order to ensure an uninterrupted simulation. A checklist was developed for each scenario to guide feedback by peer observers and case tutors.

All cases started with a presentation by the case tutor who enacted ER staff reporting a patient to the on-call physicians. Simulated Patients (SP) or simulators were placed as required by the scenario. Guideline-oriented therapy including airway-management was possible in all cases. I.v. medication and/or oxygen could be administered if required. An overview of the developed cases is provided in Table 1.

**Table 1:** Cases and simulation settings

Discipline	Diagnosis (guidelines as sources)	Mode of simulation	Anticipated course of management
Pulmology	Exacerbated COPD	SP with examination possible	Chest X-Ray, blood-gas analysis, continous monitoring
Neurology	Ischemic media-stroke	SP with examination possible	CCT, continous monitoring
Cardiology	STEMI & non-sustained ventricular tachykardia	SP with examination possible	12-channel ECG, enzymes, continous monitoring
Anaesthesia	Ventricular fibrillation following STEMI	simulator-based approach	continous monitoring, ACLS
Surgery 1	Hemodynamic instable ruptured spleen	simulator-based approach with advanced monitoring	ATLS with FAST, continous monitoring
Urology	Urinary tract infection & pregnancy	SP with examination and sonography possible	urin test, ultrasound and gynaecological referal
Surgery 2	Head laceration	SP with examination and preparation of wound possible	Stitching of the wound

### Implementation

SPs were trained for five case scenarios (pulmonology, cardiology, neurology, urology and surgery 2, see Table 1). To guarantee an appropriate level of fidelity both hybrid simulations and mechanical simulators were used [14, 19]. To represent a real time course of events, all laboratory orders and radiology inquiries had to be requested by phone and/or in written form. The operational headquarters delayed their answer depending on the requested examination. Participants finished a scenario by arranging for the patient to be transferred to a ward or to be discharged. Each scenario lasted approximately 30–45 min including feedback. Additional technical details of the simulation, a detailed description of every case and used guidelines are provided as supporting information (see Additional files 1 and 2).

### Roles of participants

For each scenario students took one of three roles:

- the *team leader* was responsible for the entire process – coordinating the team, choosing the right diagnosis and treating the patient accordingly.
- the *team member* was an active part of the group and supported the team leader throughout the process of finding the right diagnosis and treating the patient.
- the *team observer* observed the team using a checklist and provided feedback afterwards.

Roles within the group changed with each scenario so that at the end of the night each student had at least once taken on each role. Each group could freely develop their teamwork throughout the night shift. A peer tutor supervised and counseled the group.

### Multi-source feedback

We used multi-sourced feedback [20] given by observers with specific assignments:

- the *SP* focused on communication using the Calgary-Cambridge Observation Guide (CCOG) to guide his or her feedback [21, 22].
- the *team observer* gave checklist-based feedback in order to provide the team with external observations but also to increase active monitoring of the simulation for his or her personal learning effect.
- the *case tutor* focused on the decision-making process with regards to medical content using case-specific checklists.
- the *peer tutor* focused on general teamwork and the development of team dynamics and gave feedback in a distinct setting to separate it from the case scenarios.

All tutors are trained in giving feedback and have extensive experience in peer teaching. Participants had experience giving and receiving feedback through curricular events. All SPs are trained regularly.

### Statistical analysis

Collected data were analyzed with IBM SPSS Statistics 21.0 (SPSS Inc, Chicago, IL, USA). All data were first analyzed descriptively (mean, standard deviation). Confounder for feeling of preparedness were analyzed with Mann-Whitney-U-Tests, for differences between feeling of preparedness 1 and feeling of preparedness 2 we used a related sample Wilcoxon signed-rank test. For analysis of role and confidence we conducted a repeated measures analysis of variance (ANOVA), results of which we report as *F*- and *p*-values. Correlations between roles were analyzed with Pearson-correlations. Significance was defined as *p* < 0.05, Cohen’s *d* was calculated as effect size. We further used G*Power, version 3.1.9.2 [23] to calculate the power achieved. We determined a gain of 0.51 on the Likert scale used from before to after the simulation as the smallest meaningful difference, because such a change would imply that participants chose one point better on the scale slightly more often than expected by chance. The primary dataset is provided as supporting information (see Additional file 3).

## Results

A total of 30 students (20 female) participated in the simulation. Three participants had previous medical experience as a paramedic (2) or nurse (1). All 30 available places were booked up after 30 min in the online registration.

### Feeling of preparedness

Participants feel rather ill prepared to care for patients before the simulation regardless of specialty (Mean −0.34) with no significant differences between gender (*p* = 0.075) or age (*p* = 0.9).

Right after each case students feel confident in their actions and with how they handled the case (Mean 0.95). All participants completed all surveys during the event (100 % response rate), 18 of the 30 participants (60 %) completed the online survey five days after the simulation and showed a significant increase in their general feeling of preparedness compared to before the simulation (*p* = 0.001). Participants now report to generally feel prepared (Mean 0.66); the effect is large (*d* = 1.86).

We analyzed these overall effects for every implemented discipline during simulation and found significant increases in the feeling of preparedness in anesthesiology, urology and taking history (see Table 2). The power of this study to detect a change in feeling of preparedness of 0.51 or greater was 99.79 %.

**Table 2:** Feeling of preparedness and change from before to five days after simulation

Discipline	Feeling of prepardness Baseline (Mean & SD)	Feeling of prepardness Follow Up (Mean & SD)	*p*-value
Overall	−0.34 (0.49)	0.66 (0.59)	0.001**
Taking History	1.27 (1.02)	1.72 (0.9)	0.035*
Anaesthesiology	0.14 (1.06)	1.17 (0.62)	<0.001***
Urology	−0.77 (1.25)	0.28 (1.53)	0.013*
Cardiology	−0.1 (1.06)	0.28 (1.13)	0.145
Pulmonology	−0.4 (0.97)	0.11 (1.13)	0.07
Surgery	0.13 (1.33)	0.83 (1.3)	0.101
Neurology	−0.47 (1.07)	0.22 (1.11)	0.1

Likert scales from +3 (totally agree) to −3 (totally disagree) we used for each item. *p < 0,05, **p = 0,001, ***p < 0,001

### Role and confidence

In a repeated measures ANOVA with case as the within subject and role as the between subject factor, the self-reported confidence of participants is independent of their role during the simulation (F(2,52) = 0.123 *p* = 0.884). Both, team members and observers, are equally capable of judging the team leader’s confidence independent of their own role (F(2,52) = 2.055 *p* = 0.138). How an active team member judges the team leader’s confidence is in part predicted by his or her own confidence (*r* = 0.61; *p* < 0.001) while the confidence of the team leader judged by the passive observers does not correlate to the observer's personal confidence (*r* = 0.188; *p* = 0.32).

### General evaluation

The simulation was evaluated very positively. Students were especially satisfied with how their peer tutors cared for them (Mean 2.93), how the SPs portrayed the patients, the difficulty of the scenarios and their opportunity to apply knowledge learned in medical school (all Mean > 2.7). The quality of the simulation was judged as very good (Mean 2.58). The ratings of each scenario right after the case correspond to the overall evaluation of the night shift.

Students reported to take most out of the feedback given by the case tutors (Mean 2.5) and slightly less out of the feedback by SPs and observing team members (both Mean 2.0).

## Discussion

In line with previous findings [11, 24], especially in acute care [2], this study identifies a low feeling of preparedness among medical school graduates with results comparable to previous German [10] and British [3] studies. Our results provide evidence that even a relatively short simulation lasting just one night is effective in increasing students self-efficacy significantly as we observed an overall effect size of *d = 1.86.* Cohen himself suggested to classify effects as small when *Cohen's d > 0.2*, as medium when *d > 0.5* and as large when *d > 0.8* [25].

Intentionally including phases with observational tasks instead of active participation into the simulation may very well explain the simulations large increase simulation efficiency. Stegmann et al. previously demonstrated that hands-on-learning is as efficient as vicarious learning in the acquisition of complex manual skills [16] and Bloch and Bloch successfully used this method in ER-training sessions [26]. Active observation however is a requirement for vicarious learning [27] and giving feedback further enhances it [28]. Our results show that the effect of vicarious learning extends beyond knowledge and skills acquisition and affects situational confidence and ultimately the feeling of preparedness which we found to be unrelated to a learner’s role during simulation. This provides a convenient opportunity for educators to increase group size in simulation with distributed, changing roles among participants and can influence the ratio of staff vs. participants to a more economic one. Furthermore, a recent study has demonstrated a large increase in diagnostic accuracy if patients are diagnosed by teams instead of individuals [29], further increasing the necessity to train medical staff in collaboration and to improve familiarity between ER-teammates, which was found to be surprisingly low in a recent observational study [30].

Training in the night may also be beneficial – nighttime hours are a neglected part of physicians training and may help to better prepare medical graduates for clinical settings [31] and reduce subjective stress of residents working on nighttime [24].

The observation that students significantly gained confidence in history taking may be explained by the facts that a) history taking was required in all cases presented during the night shift and students thus had ample opportunity to practice and b) history taking is directly observable to fellow students and tutors and participants may thus have received plenty of feedback regarding their interviewing skills. We can however only speculate as to why students feeling of preparedness improved for some (i.e. anesthesiology and urology) but not other (i.e. cardiology, pulmonology, surgery and neurology) disciplines and reasons might be discipline-specific. The change in urology may well be attributed to the fact that students hear little to nothing about this discipline during their course of studies [17, 18], while the increased feeling of preparedness in anesthesiology may be due to the high prevalence of algorithms in this discipline. However, the factors that determine changes in the feeling of preparedness warrant further study.

Beyond their implication for simulation practice, our results may also effect future studies of physician confidence. The observation that a team leaders self-reported confidence is not significantly different from his or her confidence judged by observers indicates an equivalence of self-reported and behavioral indicators of situational confidence. This finding further justifies the use of both measures in research on situational confidence, elsewhere termed self monitoring [32]. How the previous experimental finding, that discrepancies in confidence between team members is predictive of team failure [30], translates to real-world medical practice is currently explored in different studies [33]. Although we also did not find differences between team leader’s confidence and their confidence judged by team members, team members account of the leader’s confidence correlates to their own and should thus not be regarded as a valid measure.

### Limitations

Because of the high personal effort and costs per participant, only a small number of students were included into the night shift simulation and this pilot study. This might be one reason for non-significant changes in feeling of preparedness in some disciplines. Achieved power however was adequate, thus implying that increasing sample size would likely only lead to the identification of irrelevant findings.

Further, one could argue that the feeling of preparedness is not necessarily linked to objective performance [34], an aspect discussed controversially [35] since self-efficacy is known to become a self-fulfilling prophecy by actually raising the chances of success on a given task [36]. In line with this model of self-efficacy, Bloch [26] and Schubert [37] both found good performance to be associated with high levels of self-reported feelings of preparedness.

## Conclusion

Best-practice simulation increases the feeling of preparedness in medical students but remains expensive in the conceptual process. Assigning participants to different roles during simulation is a convenient way to increase group size. These roles have no negative influence on the increase in self-efficacy and provide an opportunity for implementing multi-source peer-feedback. The feeling of preparedness of the active team members and leader also is apparent to observers and can be used as part of a debriefing after a simulation.

## Abbreviations

ANOVA, analysis of variance; CCOG, Calgary-Cambridge observation guide; ER, emergency room; SP, simulated patient

## Additional files

Additional file 1: Case descriptions (used Guidelines indicated as references). (PDF 87 kb) Additional file 2: Technical details. Diagnosis and technical details for every case. (PDF 70 kb) Additional file 3: Dataset of the study. (XLS 65 kb)
