# Supplementary material for: A simulated night shift in the emergency room increases students’ self-efficacy independent of role taking over during simulation
Source: BMC Med Educ. 2016 Jul 15;16:177. doi: 10.1186/s12909-016-0699-9 (PMC4946185; doi:10.1186/s12909-016-0699-9)
Supplement: Additional file 1: — Case descriptions (used Guidelines indicated as references). (PDF 87 kb) [file 12909_2016_699_MOESM1_ESM.pdf]

A Simulated Night Shift in the Emergency Room Increases Students' Self-efficacy Independent of Role Taking over during Simulation

F Stroben, T Schroeder, KA Dannenberg, A Thomas, A Exadaktylos, WE Hautz

Additonal File 1. Case-Description (used guidelines indicated as references).

Pulmonology (1,2):

A 65-year-old male with known COPD presents with mild fever and productive coughing over the last week and increasing, now acute dyspnea. The uneasy patient is noncompliant about his existing therapy. Physical examination shows elevated temperature, silent lungs on auscultation and a prolonged exspiration. Projected outcome of the simulation is the decision to hospitalize the patient for treatment of an exacerbated COPD.

Neurology (3):

A 58-year-old female presents with loss of speech and weakness. The patient is anxious and has a history of atrial fibrillation with anticoagulation treatment. Neurologic examination reveals broca aphasia and hemiplegia. CCT should be conducted and shows an ischemic stroke of the middle cerebral artery. Lysis is contraindicated because of the patients current medication and the patient has to be transferred to a stroke-unit.

Cardiology (4,5):

A 50-year-old troubled male presents with acute chest pain radiating into his left arm since 30 minutes. He is short of breathing, has cold sweat and is seriously worried. ECG shows an ST-segment-elevation in II, III and avF. The patient has several risk factors for coronary heart disease but no previous AP episode. Over the course of the case, the patient develops non-sustained ventricular tachycardia. An in-house catheter intervention is not available so the patient has to be relocated by a mobile intensive care unit (mICU) for treatment of a ST-elevation myocardial infarction.

Anesthesiology (6):

The 50-year-old patient with STEMI and symptoms previously described (see Cardiology case) is being relocated by a mICU to another hospital. On the way, the patient becomes unconscious and ECG shows ventricular fibrillation. After performing ACLS the patient has a return of spontaneous circulation and can be transferred successfully to receive a coronary intervention.

Surgery 1 (7):

A 32-year-old male hospital employee fell down from a ladder at work and is found conscious under a shelf, which dropped on his belly. He presents heavy abdominal pain and GCS decreases over the course of the case. The team has to perform ATLS on-site, transfer the patient to the ER and continue ATLS. The case tutor is available as consultant in the trauma room. FAST-sonography shows a ruptured spleen with serious blood loss. The patient has to be transferred for surgery after circulation has been stabilized.

Urology (8):

A 25-year-old female presents with dysuria, more frequent urination, morning sickness and amenorrhea. The patient seems carefree and doesn't know she is pregnant. Urin alysis is available and reveals a urinary tract infection. A gynecological consultation and ultrasound are available. After diagnosing and starting treatment, the patient can be discharged.

Surgery 2 (9):

A 20-year-old intoxicated male presents with a laceration on his forehead and bruised legs and arms following a bike accident. He has a minor alcohol-intoxication but reacts adequately, has not lost consciousness at any time and has a GCS of 15. Physical and neurologic examination show no sign of fracture or intracranial hemorrhage. No minor or major CHIP-criteria are fulfilled and CCT isn't indicated. After receiving sutures, the patient is released with a friend in charge.

Used Guidelines

1. Vogelmeier C, Buhl R, Gillissen A, Kardos P, Magnussen H, Morr H, et al. Guidelines for the Diagnosis and Therapy of COPD Issued by Deutsche Atemwegsliga and Deutsche Gesellschaft für Pneumologie und Beatmungsmedizin. Pneumologie. 2007;61:e1–40.

2. Qaseem A, Snow V, Shekelle P, Sherif K, Wilt TJ, Weinberger S, et al. Diagnosis and management of stable chronic obstructive pulmonary disease: a clinical practice guideline from the American College of Physicians. Ann Intern Med. 2007;147(9):633–8.

3. Jauch EC, Saver JL, Adams HP, Bruno A, Connors JJB, Demaerschalk BM, et al. Guidelines for the early management of patients with acute ischemic stroke: a guideline for healthcare professionals from the American Heart Association/American Stroke Association. Stroke. 2013 Mar;44(3):870–947.

4. Steg PG, James SK, Atar D, Badano LP, Blömstrom-Lundqvist C, Borger M a, et al. ESC Guidelines for the management of acute myocardial infarction in patients presenting with ST-segment elevation. Eur Heart J. 2012 Oct;33(20):2569–619.

5. O'Gara PT, Kushner FG, Ascheim DD, Casey DE, Chung MK, de Lemos J a, et al. 2013 ACCF/AHA guideline for the management of ST-elevation myocardial infarction: executive summary: a report of the American College of Cardiology Foundation/American Heart Association Task Force on Practice Guidelines. Circulation. 2013 Jan 29;127(4):529–55.

6. Nolan JP, Soar J, Zideman D a, Biarent D, Bossaert LL, Deakin C, et al. European Resuscitation Council Guidelines for Resuscitation 2010 Section 1. Executive summary. Resuscitation. 2010 Oct;81(10):1219–76.

7. Schoeneberg C, Schilling M, Burggraf M, Fochtmann U, Lendemans S. Reduction in mortality in severely injured patients following the introduction of the "treatment of patients with severe and multiple injuries" guideline of the German society of trauma surgery - a retrospective analysis of a level 1 trauma center (2010-2. Injury. Elsevier Ltd; 2014 Mar;45(3):635–8.

8. Schnarr J, Smaill F. Asymptomatic bacteriuria and symptomatic urinary tract infections in pregnancy. Eur J Clin Invest. 2008 Oct;38 Suppl 2:50–7.

9. Smits M, Dippel DWJ, Steyerberg EW, de Haan GG, Dekker HM, Vos PE, et al. Predicting intracranial traumatic findings on computed tomography in patients with minor head injury: the CHIP prediction rule. Ann Intern Med. 2007;146(6):397–405.
