# Supplementary material for: A simulated night shift in the emergency room increases students’ self-efficacy independent of role taking over during simulation
Source: BMC Med Educ. 2016 Jul 15;16:177. doi: 10.1186/s12909-016-0699-9 (PMC4946185; doi:10.1186/s12909-016-0699-9)
Supplement: Additional file 2: — Technical details. Diagnosis and technical details for every case. (PDF 70 kb) [file 12909_2016_699_MOESM2_ESM.pdf]

A Simulated Night Shift in the Emergency Room Increases Students' Self-efficacy Independent of Role Taking over during Simulation

F Stroben, T Schroeder, KA Dannenberg, A Thomas, A Exadaktylos, WE Hautz

Additional File 2. Technical details.

Discipline	Diagnosis	Mode of simulation	Available medical equipment	Anticipated course of management
**Pulmology**	Exacerbated COPD	SP, history taking & examination possible	PC-based monitoring (Vital Sign Simulator, healthysimulation.com), arterial puncture wrist (Kyoto Kagaku Co. Ltd, Kyoto, Japan), 12-channel ECG, defibrillator, infusion sets, i.v. medication, oxygen-therapy	Chest X-Ray, blood-gas analysis, continous monitoring
**Neurology**	Ischemic media-stroke	SP, history taking & examination possible	PC-based monitoring, arterial puncture wrist, infusion sets, i.v. medication, oxygen-therapy	CCT, continous monitoring
**Cardiology**	STEMI & non-sustained ventricular tachykardia	SP, history taking, examination and 12-channel ECG on patient possible	PC-based monitoring, arterial puncture wrist, 12-channel ECG, defibrillator, infusion sets, i.v. medication, oxygen-therapy	12-channel ECG, enzymes, continous monitoring
**Anaesthesia**	Ventricular fibrillation following STEMI	Laerdal-ALS-Simulator with SimPad™ (Laerdal Medical GmbH, Puchheim, Germany)	defibrillator with monitoring, ambulance vehicle, infusion sets, i.v. medication, oxygen-therapy, ventilator	continous monitoring, ACLS
**Surgery 1**	Hemodynamic instable ruptured spleen	Laerdal-ALS-Simulator with SimPad™ and SimPad™ Patient Monitor (Laerdal Medical GmbH, Puchheim, Germany)	SimPad™ based monitoring, ultrasound-simulator (Sonofit GmbH, Darmstadt, Germany), ventilator, arterial puncture wrist, 12-channel ECG, sonography, defibrillator, infusion sets, i.v. medication, oxygen-therapy, endotracheal intubation	ATLS with FAST, continous monitoring
**Urology**	Urinary tract infection & pregnancy	SP, history taking, examination and sonography possible	sonography, urine test, infusion sets, i.v. medication,	urin test, ultrasound and gynaecological referal
**Surgery 2**	Head laceration	SP, history taking, examination, and preparation of wound possible	hybrid suture simulation and devices, infusion sets, i.v. medication	Stitching of the wound

Diagnosis and technical details for every case.
